# Discrete Dynamic Model of the Mammalian Sperm Acrosome Reaction: The Influence of Acrosomal pH and Physiological Heterogeneity

**DOI:** 10.3389/fphys.2021.682790

**Published:** 2021-07-19

**Authors:** Andrés Aldana, Jorge Carneiro, Gustavo Martínez-Mekler, Alberto Darszon

**Affiliations:** ^1^Departamento de Genética del Desarrollo y Fisiología Molecular, Instituto de Biotecnología, Universidad Nacional Autónoma de México, Cuernavaca, Mexico; ^2^Centro de Ciencias de la Complejidad, Universidad Nacional Autónoma de México, Mexico City, Mexico; ^3^Instituto Gulbenkian de Ciência, Oeiras, Portugal; ^4^Instituto de Tecnologia Química e Biológica António Xavier, Universidade Nova, Oeiras, Portugal; ^5^Instituto de Ciencias Físicas, Universidad Nacional Autónoma de México, Cuernavaca, Mexico

**Keywords:** regulatory network, dynamic model, discrete dynamics, acrosome reaction, sperm signaling pathway, pH regulation, physiological heterogeneity, mammalian fertilization

## Abstract

The acrosome reaction (AR) is an exocytotic process essential for mammalian fertilization. It involves diverse physiological changes (biochemical, biophysical, and morphological) that culminate in the release of the acrosomal content to the extracellular medium as well as a reorganization of the plasma membrane (PM) that allows sperm to interact and fuse with the egg. In spite of many efforts, there are still important pending questions regarding the molecular mechanism regulating the AR. Particularly, the contribution of acrosomal alkalinization to AR triggering physiological conditions is not well understood. Also, the dependence of the proportion of sperm capable of undergoing AR on the physiological heterogeneity within a sperm population has not been studied. Here, we present a discrete mathematical model for the human sperm AR based on the physiological interactions among some of the main components of this complex exocytotic process. We show that this model can qualitatively reproduce diverse experimental results, and that it can be used to analyze how acrosomal pH (pH_*a*_) and cell heterogeneity regulate AR. Our results confirm that a pH_*a*_ increase can on its own trigger AR in a subpopulation of sperm, and furthermore, it indicates that this is a necessary step to trigger acrosomal exocytosis through progesterone, a known natural inducer of AR. Most importantly, we show that the proportion of sperm undergoing AR is directly related to the detailed structure of the population physiological heterogeneity.

## 1. Introduction

The acrosome reaction (AR) is an exocytotic process in sperm that is essential for fertilization in many species, including mammals. It involves multiple and complex biochemical, biophysical, and morphological changes, which we will refer to in the rest of this paper as physiological changes that culminate in the release of different hydrolytic enzymes to the extracellular medium, as well as reshaping of the plasma membrane (PM).

The AR confers sperm the ability to interact and fuse with the egg (Inoue et al., 2011; Jin et al., 2011; Bianchi and Wright, 2016). Despite many years of research, the natural stimuli of this fundamental process are still not completely elucidated. Furthermore, whether the hydrolytic enzymes released to the extracellular medium facilitate the passage of sperm through the zona pellucida remains an open question in many species (Darszon et al., 1999; Florman and Ducibella, 2006; Wassarman and Litscher, 2009; Jin et al., 2011; Okabe, 2016). The specific physiological events that trigger the AR remain elusive, although we know that in the course of this process there is an increase in the intracellular concentration of Ca^2+^ ([Ca^2+^]_*i*_) (Darszon et al., 1999; Sánchez-Cárdenas et al., 2014; Stival et al., 2016) as well as an increase in intracellular pH (pH_*i*_) (Nishigaki et al., 2014; Stival et al., 2016). The regulation of pH_*i*_ is important for the functioning of a variety of proteins. The sperm exclusive Ca^2+^ channel (CatSper) and K^+^ channel (Slo3) are strongly pH_*i*_ dependent (Chávez et al., 2014; Nishigaki et al., 2014; Zhang et al., 2006). The rise in intra-acrosomal pH (pH_*a*_) can lead to increases in [Ca^2+^]_*i*_ and spontaneous AR (Nakanishi et al., 2001; Chávez et al., 2017) both in mouse and human sperm (Chávez et al., 2017). However, the relevance of acrosomal alkalinization under a naturally triggered exocytotic process is not well understood. It is known that Pg promotes [Ca^2+^]_*i*_ elevation by stimulating CatSper in human sperm (Baldi et al., 2009; Miller et al., 2016).

Cell population studies indicate that only a fraction of sperm is capable of undergoing AR, either spontaneously or after induction with progesterone (Pg), a known AR inducer present in the female tract at the relevant concentration. In human and mice sperm samples, 15–20% of cells undergo spontaneous AR (Nakanishi et al., 2001), whereas only 20–30% undergo Pg-induced AR (Stival et al., 2016). Although this suggests that physiological heterogeneity plays a role in determining the proportion of sperm capable of undergoing AR either spontaneously or after Pg induction, such heterogeneity has not yet been studied. Moreover, the AR develops progressively in time (Sánchez-Cárdenas et al., 2014), implying that at a particular time, sperms are heterogeneous in their physiological states. This hypothesis is supported by reports in the literature showing a wide non uniform range of values for the concentration of distinct intracellular components in a sperm population for different species (Luque et al., 2018; Balbach et al., 2020a; Molina et al., 2020).

In the present work, we implement a generalization of the Gene Regulatory Network as formalized by Stuart Kauffman in 1969 (Kauffman, 1969) and used in many different systems (Mendoza and Alvarez-Buylla, 1998, 2000; Espinal et al., 2011; Yang et al., 2018) to construct a mathematical and computational model that represents the main physiological interactions involved in AR. We show that this model can qualitatively reproduce many of the experimental results reported in literature and we use it to characterize how the physiological heterogeneity in a sperm population affects the proportion of cells capable of displaying spontaneous and Pg-induced AR.

Our model also corroborates that acrosomal alkalinization can trigger AR by itself in a fraction of the sperm population and suggests that it is important for AR induction by Pg in another fraction. Together, our results indicate that physiological heterogeneity is closely related with the proportion of sperm capable of displaying AR, and that a pH_*a*_ increase is an essential event in the process of the AR.

## 2. Regulatory Network Background

### 2.1. AR Preconditions

Capacitation is a precondition necessary for natural AR in mammalian sperm. It is a complex process involving PM remodeling, cholesterol removal, extensive changes in protein phosphorylation patterns, and increases in pH_*i*_ and [Ca^2+^]_*i*_ (Bianchi and Wright, 2016), as well as membrane hyperpolarization (De La Vega-Beltran et al., 2012; Chávez et al., 2013; Stival et al., 2016). Only a subpopulation of sperm (20–40%) becomes capacitated, and the mechanism explaining how this subpopulation is selected are far from clear.

Cell pH_*i*_ regulation is performed mainly by H^+^ fluxes between the extracellular medium, the cytosol, and internal stores, as well as HCO_3_^-^ transport and metabolism (Nakanishi et al., 2001; Lishko et al., 2012; Romero et al., 2013; Nishigaki et al., 2014; Chae et al., 2017; Soriano-Úbeda et al., 2019; Balbach et al., 2020a; Hidalgo et al., 2020). In the PM, a Na^+^/HCO_3_^-^ cotransporter (NBC) allows HCO_3_^-^ uptake (Romero et al., 2013; Nishigaki et al., 2014) while a pH-dependent Cl^−^/HCO_3_^-^ exchanger (SLC) extrudes it to the extracellular space. Increases in HCO_3_^-^ intracellular concentration ([HCO_3_^-^i) also activate a soluble Adenilate Cyclase (sAC) (Okamura et al., 1985; Chen et al., 2000; Kleinboelting et al., 2014; Nishigaki et al., 2014). The sperm-specific Na^+^/H^+^ exchanger (sNHE) contributes importantly to cytosolic pH_*i*_ regulation in mouse sperm (Nakanishi et al., 2001; Wang et al., 2007; Nishigaki et al., 2014), although recent results indicate it is NHA_1_ that is important for the ZP-induced mouse sperm pH_*i*_ increase during AR (Balbach et al., 2020b). In human sperm, the *H*^+^ channel Hv1 appears to be the main pH_*i*_ regulator (Lishko et al., 2012; DeCoursey, 2013; Nishigaki et al., 2014; Chae et al., 2017; Miller et al., 2018).

The intra-acrosomal space is maintained acidic (pH_*a*_~ 5.4) principally by a H^+^ V-ATPase in the acrosomal membrane (Nakanishi et al., 2001; Sun-Wada et al., 2002; Chávez et al., 2017). In turn, the acidic pH_*a*_ contributes to cytosolic acidification by means of a nonspecific acrosomal pH_*a*_-dependent H^+^ outward current (HLeak_*a*_) (Nakanishi et al., 2001; Chávez et al., 2017). It has been proposed that a somatic Na^+^/H^+^ exchanger in the acrosome membrane could participate in this flux (Nakamura et al., 2005; Oberheide et al., 2017), however its existence has not been established. During capacitation pH_*a*_ increases and this elevates spontaneous AR (Nakanishi et al., 2001). Because of this, it was proposed that an increase in pH_*a*_ during capacitation may be a requirement to prepare sperm for the AR. In this direction, permeable weak bases able to alkalize the acrosome can release Ca^2+^ from acidic compartments including the acrosome, increase [Ca^2+^]_*i*_, and induce AR (Chávez et al., 2017). On the other hand, it has been proposed that acrosomal alkalinization is involved also in acrosome swelling and outer acrosomal membrane (OAM) deformation during AR by means of pH_*a*_-dependent activation of proteolytic enzymes that destabilize the acrosomal matrix (Guyonnet et al., 2014; Chávez et al., 2017).

#### Membrane Potential Regulation

There is evidence that membrane hyperpolarization is important for capacitation and therefore necessary for AR to occur. Also, a depolarization in capacitated sperm can modulate AR Darszon et al. (2011); De La Vega-Beltran et al. (2012). These events suggest the existence of a fine membrane potential (Em) regulation during capacitation and AR. Em regulates and is regulated by different ionic transporters. Membrane depolarization activates voltage-dependent channels like classical Ca_v_s (Darszon et al., 1999; Zhang and Gopalakrishnan, 2005), a controverted subject, CatSper (Chávez et al., 2014), K^+^ channels (IKsper) (Zhang et al., 2006; Chávez et al., 2014), and Hv1 (Lishko et al., 2012; Chae et al., 2017). Em also influences sNHE activity (in mouse sperm) that is activated by hyperpolarization and participates in sperm alkalinization (Chávez et al., 2014). Cationic efflux carried by IKsper and Hv1 promotes hyperpolarization, as well as HCO_3_^-^ entry by NBC activation. Finally, Ca^2+^ uptake through CatSper and possibly by Ca_v_s, and store operated Ca^2+^ channels (SOCs), aside from increasing [Ca^2+^]_*i*_ depolarizes Em (Darszon et al., 2011; Correia et al., 2015; Sosa et al., 2016).

### 2.2. The Acrosome Reaction

The acrosome is an acidic vesicle of lysosomal/Golgi origin located at the apical part of the sperm head that accumulates Ca^2+^ in its interior. It contains hydrolytic enzymes released through exocytosis to the extracellular medium when the AR occurs (Dan, 1952, 1954; Darszon et al., 2011). Although the specific sequence of events that trigger the AR after capacitation is not fully understood, there is evidence that it involves elevations of pH_*i*_ and [Ca^2+^]_*i*_, and acrosomal Ca^2+^ release (Darszon et al., 2011). These events promote and culminate in acrosome swelling, deformation of the outer acrosomal membrane (OAM), interaction and docking with the PM and finally, fusion between OAM and PM that promotes exocytosis (Mayorga et al., 2007; Sosa et al., 2014).

#### Cytosolic and Acrosomal Ca^2+^ Regulation

It is well established that orchestrated [Ca^2+^]_*i*_ elevations are crucial for the AR. In the sperm head, upon AR induction, Ca^2+^ enters the cytosol mainly through SOCs in the PM, as indicated by indirect evidence (Sosa et al., 2016). Release of inositol 3-phosphate (IP_3_) caused by phospholipase C delta 4 (PLC_δ_) activates IP_3_ receptors (IP_3_R) in the OAM (Fukami et al., 2001, 2003; Mayorga et al., 2007; Darszon et al., 2011; Tomes, 2015; Sosa et al., 2016). The latter is a [Ca^2+^]_*i*_ and IP_3_-dependent Ca^2+^ channel with one binding site for IP_3_ and two binding sites for Ca^2+^ of low and high affinity that promote opening or inactivation of the channel, respectively (Bezprozvanny et al., 1991; Foskett et al., 2007; Schmeitz et al., 2013; Belmonte et al., 2016). In the presence of IP_3_, a moderate increase in [Ca^2+^]_*i*_ results from IP_3_ receptors (IP_3_R) opening while high concentrations block the channel. IP_3_R activation releases Ca^2+^ in the acrosome. This event is detected by stromal interaction molecule (STIM) proteins in the OAM (Darszon et al., 2011; Sosa et al., 2014), which in turn reorganizes and activates SOCs in the PM (Lefièvre et al., 2012; Sosa et al., 2016). In the flagellum, CatSper, a pH-dependent and mildly voltage gated Ca^2+^ channel, increases [Ca^2+^]_*i*_ concentration (Kirichok et al., 2006; Lishko et al., 2011). Two different types of Ca^2+^ ATPases help controlling Ca^2+^ elevation in the cytosol promoted by SOCs and IP_3_Rs. The PM Ca^2+^ ATPase (PMCA) in the PM extrudes Ca^2+^ to the extracellular medium (Schuh et al., 2004; Da Costa et al., 2016) and an acrosomal Ca^2+^ ATPase (ACA) contributes to maintain the high level of [Ca^2+^]_*a*_ during the final stages of the AR. There is debate on which type of Ca^2+^ ATPase is contributing to Ca^2+^ mobilization from the acrosome. The secretory pathway Ca^2+^ ATPase pump type 1 (SPCA1) is expressed in human sperm, although it seems to be localized mainly in the neck region (Harper et al., 2005). Also, a different study shows that the sarco-/endoplasmic reticulum Ca^2+^ ATPase type 2 (SERCA2) is expressed in the acrosome and mid piece region (Lawson et al., 2007). The contribution and presence of these two forms of Ca^2+^ ATPase in the AR are yet to be fully elucidated. Ca^2+^ modulates diverse signaling pathways: activation of PLC that produces diacylglycerol (DAG) and IP_3_ (Darszon et al., 2011; Nahed et al., 2016), activation of AC that increases cyclic adenosine monophosphate (cAMP) levels as well as phosphodiesterases (PDE) that create the opposite effects (Jungnickel et al., 2007; Sosa et al., 2016), and activation of synaptotagmin (SYT) (Hutt et al., 2005), a Ca^2+^ sensor important in the final stages of membrane fusion (Mayorga et al., 2007).

#### Triggering Membrane Fusion

Fusion between OAM and MP during AR has not been fully characterized. However, several proteins involved in other exocytotic processes have been found in sperm, such as the Ras-related protein Rab3A (Iida et al., 1999; Ward et al., 1999; Yunes et al., 2000), N-ethylmaleimide-sensitive factor (NSF) (Michaut et al., 2000; Ramalho-Santos and Schatten, 2004), α-soluble NSF attachment protein (αSNAP) (Tomes et al., 2005), SNAP receptor family members (SNARE's) (Ramalho-Santos et al., 2000; Schulz et al., 1998; Tomes et al., 2002), SNARE's associated proteins like complexin (Redecker et al., 2003; Zhao et al., 2007), Ca^2+^ regulated proteins like SYT (Ramalho-Santos et al., 2000; Hutt et al., 2002, 2005) and calmodulin (Trejo and Mújica, 1990) as well as cellular transport-related proteins like dynamin (Mayorga et al., 2007; Zhao et al., 2007). Based on multiple literature reports, a model has been developed to describe the specific physiological events involved in membrane fusion during AR (Mayorga et al., 2007). This model is also consistent with experimental observations in human sperm and we take this as a starting hypothesis for our work. It considers that at early fusion stages SYT is inactive and there are inactive *cis*-SNARE complexes assembled between the OAM and PM, which keep the AR in standby until other events trigger it. Further in time IP_3_R channels open and release [Ca^2+^]_*a*_ from the acrosome, which promotes SOC channel aperture and an increase in [Ca^2+^]_*i*_. This stimulates the activity of soluble and/or transmembrane AC increasing cAMP levels (Baxendale and Fraser, 2003; Branham et al., 2006; Steegborn, 2014). There is evidence that cAMP promotes deformation and swelling of the acrosome, possibly activating Rab3A through exchange proteins directly stimulated by the rise in this messenger (EPAC), given the rise in cAMP concentration (Yudin et al., 1988; Mayorga et al., 2007). The events connecting [Ca^2+^]_*i*_ with cAMP, EPAC, and Rab3A are not clear but we consider in our network the proposal that after activation of Rab3A, NFS and αSNAP, *cis*-SNARE complexes disassemble and reorganize in *trans*-SNARE complexes (De Blas et al., 2002). At this stage, SYT dephosphorylates and fusion stands by until Ca^2+^ is released again from the acrosome to activate it. The model hypothesizes that the free Ca^2+^ in the cytosol is inaccessible to the small space between the OAM and the PM at the final stages of the AR, and that the opening of local IP_3_Rs provides the necessary Ca^2+^ that activates SYT and thus SNARE's, finally allowing the fusion between membranes.

### 2.3. Regulatory Network Implementation

**Figure 1:**
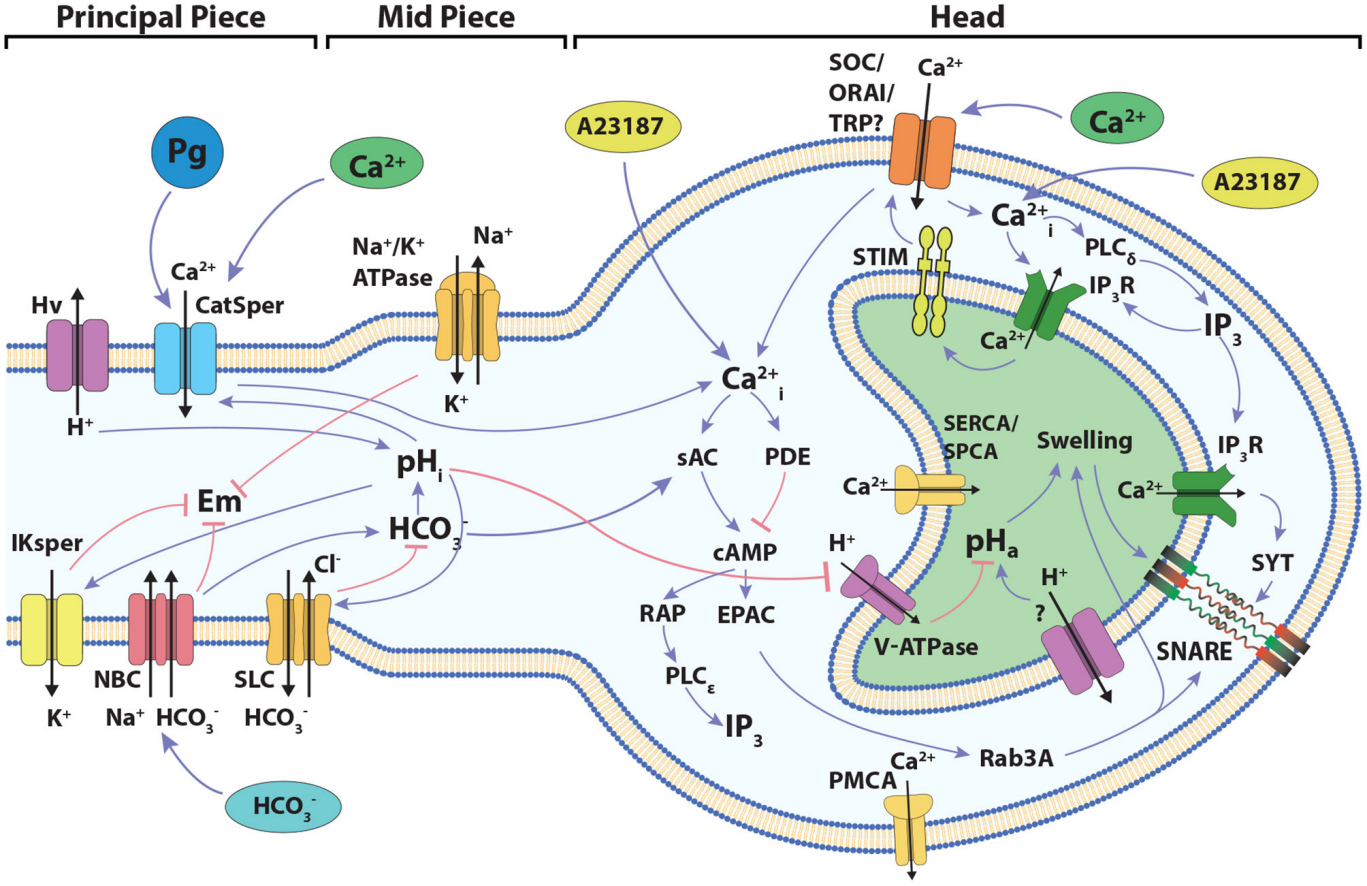
Diagram of the signaling network involved in the development of acrosome reaction in human sperm. The cartoon shows the main components and signaling events to be considered in the development of the model described in the present work. Blue links connecting components indicate positive interactions, while red links indicate negative interactions. The localization of the different components among the head and the flagellum are indicated. Progesterone activates CatSper indirectly through a lipid hydrolase (Miller et al., 2016).

**Figure 2:**
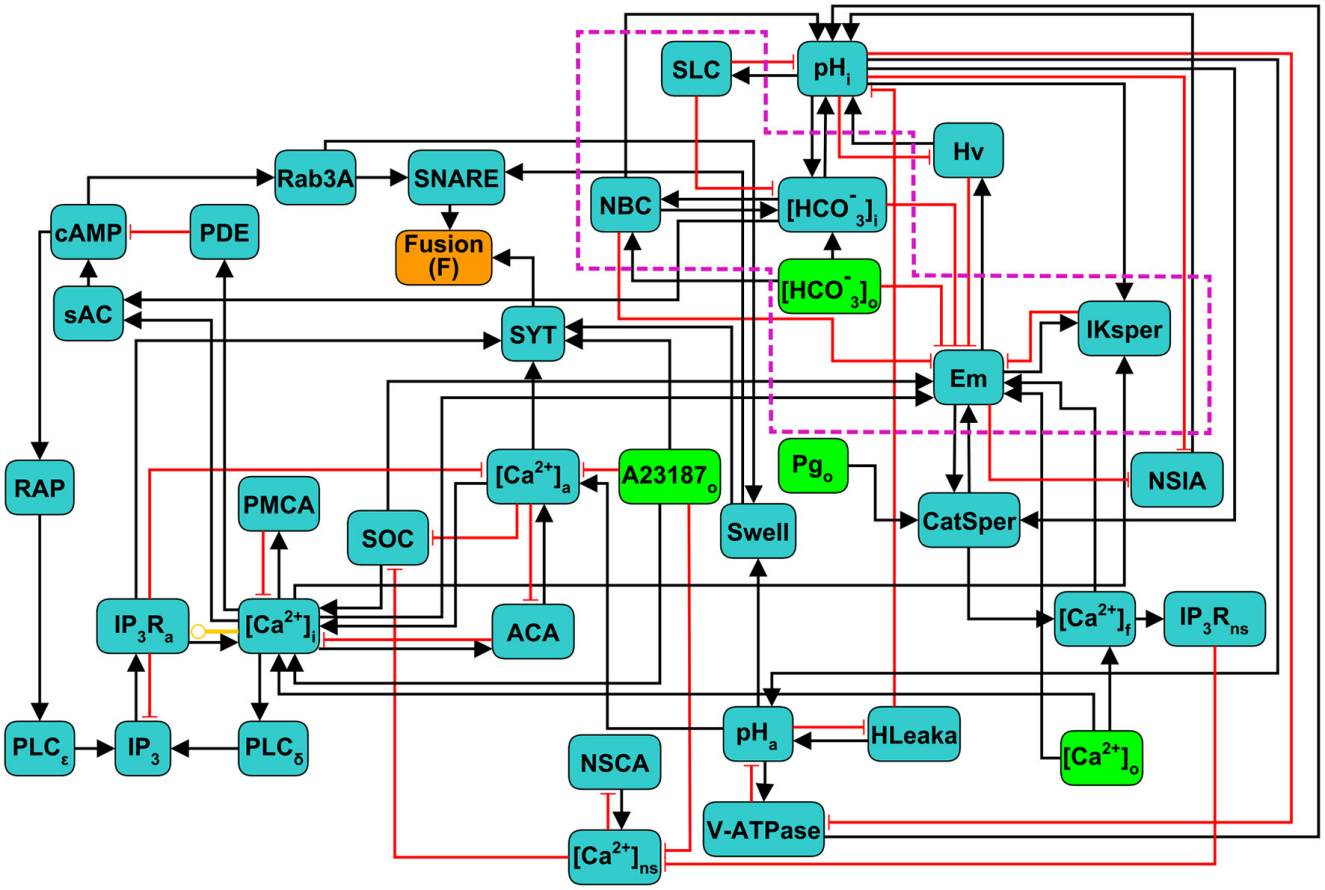
Scheme of the discrete regulatory network for acrosome reaction (AR) in human sperm. Light green nodes indicate extracellular components that can be considered as external stimuli or inputs of the network, dark green nodes represent intracellular components, the orange node Fusion (F) is the network's output reporter, representing the fusion between OAM and PM, that indicates AR completion. Black arrows indicate positive or activating interactions, red arrows indicate negative or inhibitory interactions, and yellow arrow indicates a dual interaction, depending on the values of the nodes involved. Subscripts indicate the physical location in the sperm of the physiological component represented by its corresponding node: i,a,o,f and ns represent intracellular, acrosomal, outer (extracellular medium), flagellar and neck store, respectively. Nodes inside the purple doted box are mostly involved in capacitation and are included as control variables of the necessary conditions to undergo AR.

The components and interactions of the signaling pathway involved in acrosomal reaction in human sperm, described in the previous section, are illustrated in Figure 1. This signaling pathway was translated formally into the *Regulatory Network* represented in Figure 2, consisting of 38 nodes and 87 links between nodes. In this network, each node represents one of the main components of the AR signaling pathway and links represent regulatory interactions among components. Given any two nodes A and B, their interactions can be positive, negative or dual depending on whether the activity of A promotes (black arrows) or inhibits (red arrows) the activity of B, or its action is context dependent (yellow arrow) on the activity of B. The network includes four *input* nodes that represent external stimuli, namely Ca^2+^ ionophore A23187, progesterone (Pg), and external concentrations of Ca^2+^ and HCO_3_. An *output reporter* node (Fusion or F) representing the fusion between the outer acrosomal membrane and the PM indicates the completion of the AR and is the end point of the network.

To describe and analyze the dynamical properties of this model, we use a generalization of the Boolean gene regulatory networks proposed by Stuart Kauffman in 1969 (Kauffman, 1969) that have been successfully used in different gene regulation studies (Kauffman, 1969; Mendoza and Alvarez-Buylla, 1998, 2000), as well as in biochemical regulation (Thieffry, 2007; Espinal et al., 2011; Chaouiya et al., 2012; Yang et al., 2018). In this model, the state of the whole network is described by a set of N discrete variables *x*_1_, *x*_2_, ..., *x*_*N*_, each one representing the state of one node. Most of the nodes can take values 0 or 1, corresponding to the basal and increased activity of the component, respectively. To better describe the activity and dynamics of some components, 4 nodes take more than 2 discrete values from 0, 1, 2, or 3, as shown in Table 1: Em, CatSper, [Ca^2+^]_*i*_ and pH_*i*_. The particular value of these nodes has different interpretations for each case: Em (hyperpolarized 0, equilibrium 1, mildly depolarized 2, and fully depolarized 3); CatSper (closed 0, open 1, inactivated 2); [Ca^2+^]_*i*_ (basal 0, activator 1, inhibitor 2); pH_*i*_ (acidic 0, mildly alkaline 1, fully alkaline 2). The *node state* is determined by a *regulatory function* that takes as argument the values at a particular time of its regulator nodes. Let us define *x*_*i*1_(*t*), *x*_*i*2_(*t*), …, *x*_*ik*_(*t*), the values at time *t* of the *k* regulators of node *x*_*i*_. Then the value of node *x*_*i*_ changes at each time step according to:

(1) $$x_{i}(t+1)=F_{i}(x_{i1}(t),x_{i2}(t),\ldots ,x_{ik}(t))$$

where *F*_*i*_ is the regulatory function of node *x*_*i*_. Since all nodes *x*_*i*_ are updated at each time step, this induces deterministic and synchronous dynamics. For each *F*_*i*_, the interactions with all its regulators is based on the biological knowledge described on sections 2.1 and 2.2. The list of all the regulatory functions is presented in the Supplementary Material. We also provide a concrete example of the construction of one regulatory function in Appendix A of the Supplementary Material.

**Table 1:** States and interpretation of the non-binary nodes.

**State**	**Em**	**CatSper**	**[Ca^**2+**^]_***i***_**	**pH_***i***_**
0	Hyperpolarized	Closed	Basal	Acidic
1	Equilibrium	Open	Activator	Mildly alkaline
2	Mildly depolarized	Inactivated	Inhibitor	Fully alkaline
3	Fully depolarized	N/A	N/A	N/A

*The number of states and their biological interpretation depends on how each node regulates and is regulated by other nodes. The nodes with value N/A (not applicable) in state 3 are ternary and only can take states in {0,1,2}.*

**Figure 3:**
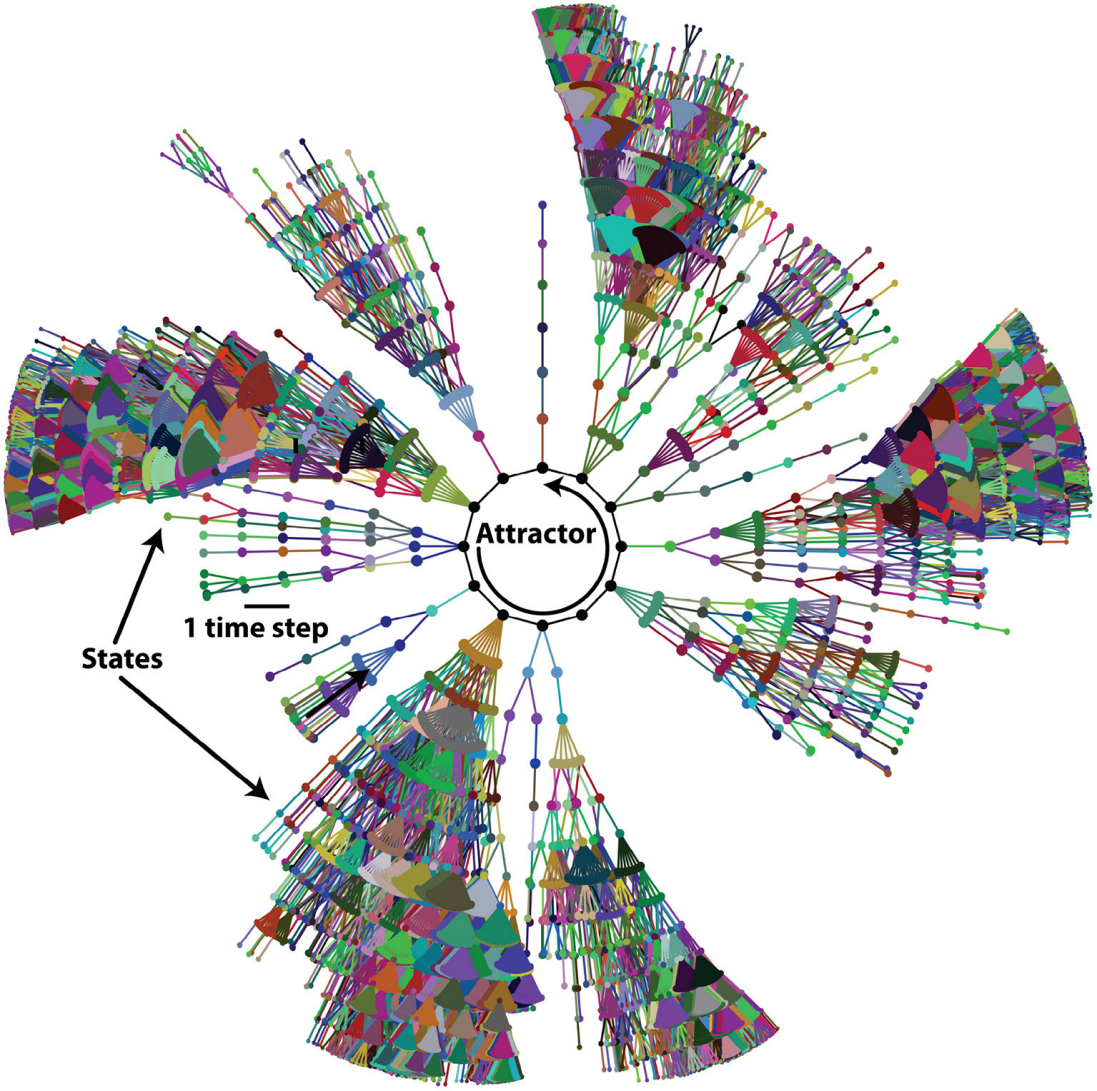
Basin of attraction of one attractor of the acrosome reaction (AR) network. Network states are symbolized by dots and links between dots represent the single-step state transitions. The network eventually reaches the attractor, symbolized by black dots at the center, connected with solid black lines. For clarity, only states at 13 or less time steps away from the attractor are shown.

The *state* of the network *X*(*t*) at time *t* is defined as the value that each variable *x*_*i*_ takes at time *t*. That is, *X*(*t*) = *x*_1_(*t*), *x*_2_(*t*), …, *x*_*N*_(*t*). Starting from any initial state of the network *X*(0) = *x*_1_(0), *x*_2_(0), …, *x*_*N*_(0), the synchronous and deterministic application of equation (1) results in one and only one successor state for *X*(*t*), which is *X*(*t*+1). Iterative application of equation (1) to calculate successor states drives the network through a series of changes that end in a so-called *attractor*, which can potentially be either a *fixed point* if the state does not change in time, or a *cyclic attractor* if a set of states are periodically visited in order. There can be many attractors for the same network. In our model, there are no fixed points and only cyclic attractors are present. All the states that under the dynamics end in a specific attractor are its *basin of attraction*. Due to the deterministic nature of the dynamics, a state can only belong to one basin of attraction. This means that the initial state of the network completely determines the attractor that the network will eventually reach. States, attractors, basins of attraction and transitions between states can be represented by a global *state transition graph*. In this graph, each node represents a state and the directed link connecting two states denotes the state transition from one to the other in a single time step. Figure 3 exemplifies a component of the state transition graph for one attractor and its basin of attraction.

It has been shown that different attractors can represent distinct functional behaviors of the system. In genetic regulatory networks, the attractors represent patterns of genetic activity that produce different phenotypes (Mendoza and Alvarez-Buylla, 1998, 2000). In our case, each state *X*(*t*) represents the physiological configuration *x*_1_, *x*_2_, ..., *x*_*N*_ of one spermatozoon at time *t* and attractors represent the different activity patterns a spermatozoon can reach.

## 3. Results

### 3.1. Attractor Space Partitions Into Three Functional Subpopulations

Imagine a population of sperm, each running internally its own regulatory network. We start each network in the population from a random initial state and let the dynamics run. Different networks will end up in different attractors depending on the initial state they started from. Thus, although all the networks in the population are identical, the final configuration of the population will be heterogeneous as different networks will end up in different attractors.

To consolidate the construction of the discrete regulatory network model of the human sperm AR, we first characterized and classified the network attractors and their corresponding basins of attraction.

**Figure 4:**
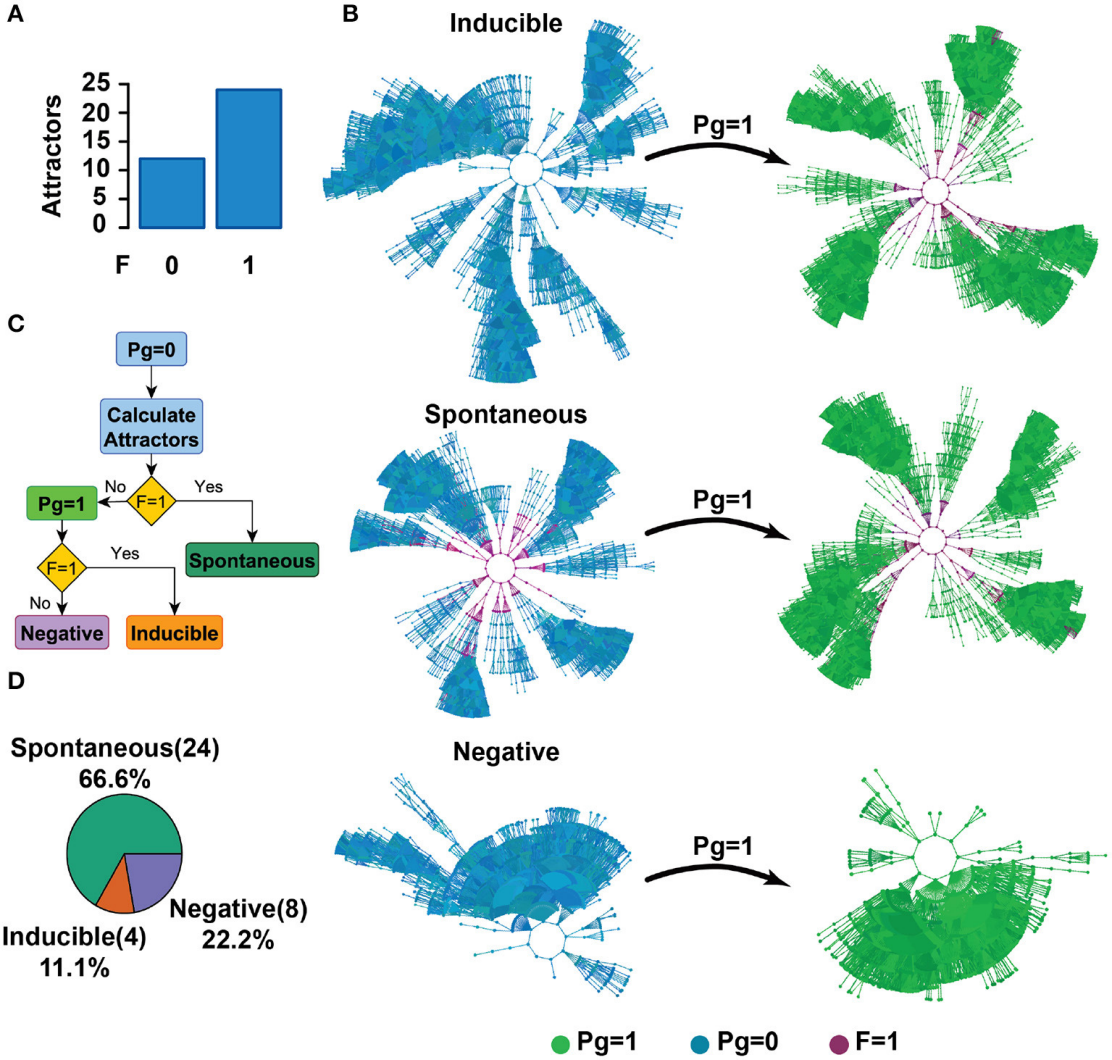
Attractor landscape partitions into three functional groups: Spontaneous, Inducible, and Negative. **(A)** The number of attractors reaching a membrane fusion state (F = 1) or lack of it (F = 0) in the absence of Pg (Pg = 0). **(B)** Diagram of the classification criteria of the attractor space in three functional groups. Colors represent the activity of the Pg node and the reporter node Fusion (F); blue dots correspond to states where Pg = 0, whereas green dots indicate Pg = 1. Purple dots represent states reaching F = 1. Considering the attractors of **(A)**, Spontaneous attractors present membrane fusion (F = 1) in the absence of Pg, in which case Pg addition does not affect the membrane fused state [these attractors correspond to the second bar of **(A)**]. From the remaining attractors that do not present membrane fusion (F = 0), Inducible attractors are those where the fusion state (F = 1) is reached only after a Pg stimulus (Pg = 1). Negative attractors never reach the fusion state (F = 1) regardless of the value of Pg. **(C)** Flow diagram of the classification criteria described in **(B)**. **(D)** Distribution of attractors in the absence of Pg (Pg = 0), classified in terms of their response to Pg.

Experimentally it has been determined that Pg released by the cumulus cells under natural conditions (Meizel et al., 1997; Baldi et al., 2009) activates CatSper and therefore increases [Ca^2+^]_i_, though other responses have been invoked in the literature (Stival et al., 2016), promoting the AR. In our model, in order to incorporate this result we calculated the network attractors and classified them into three main functional classes in terms of their biological interpretation: first, we identified all attractors in the absence of Pg and recognized those in which the reporter node Fusion is active (F = 1) (Figures 4A,C). Networks in these attractors represent sperm that will undergo membrane fusion without the Pg stimulus and correspond to the spontaneous AR; therefore, we label these attractors as Spontaneous. Next, on the remaining attractors in which the reporter node Fusion is inactive (F = 0), we activated the Pg node, simulating the effects of adding progesterone to the medium. Consequently, networks residing in one of these source attractors (Figure 4B, left column) “jump” into the basin of attraction of a different target attractor (Figure 4B, right column). If inside the target attractors the reporter node Fusion activates (F = 1), we label the source attractors as Inducible (Figures 4B,C) as networks in those attractors represent sperm that undergo the AR only after the Pg stimulus. Finally, if the reporter node fusion does not activate inside the target attractors, then the source attractors are labeled as Negative (Figures 4B,C) since networks residing in those attractors represent sperm that will not undergo the AR even after the Pg stimulus.

According to our classification criteria, Figure 4D shows the distribution of attractors before the Pg stimulus in each of these functional groups.

### 3.2. Physiological Heterogeneity and Model Validation

Human sperm seem to be heterogeneous in their capacity to undergo AR in vitro. About 15–20% of the capacitated sperm spontaneously acrosome react (Nakanishi et al., 2001) and 20–30% of the cells undergo AR in response to Pg (Stival et al., 2016). However, the relation between the physiological heterogeneity in a sperm population and the proportion of cells undergoing spontaneous and Pg-induced AR has not been established.

As in different discrete models reported in the literature (Mendoza and Alvarez-Buylla, 2000; Espinosa-Soto et al., 2004; Álvarez-Buylla et al., 2008; Espinal et al., 2011; Espinal-Enríquez et al., 2017), we used a uniformly distributed random set of network initial states to evaluate the possible fates of the network. States representing cells that have already undergone membrane fusion or that have a fusion machinery already activated were excluded from the analysis, since these nodes are mostly insensitive to any stimuli and are biologically unreasonable. Therefore, we restricted the set of initial states to those where the values of the reporter nodes, Fusion, Swell, and SNARE nodes, were 0. The values for all the other nodes were assigned randomly.

**Figure 5:**
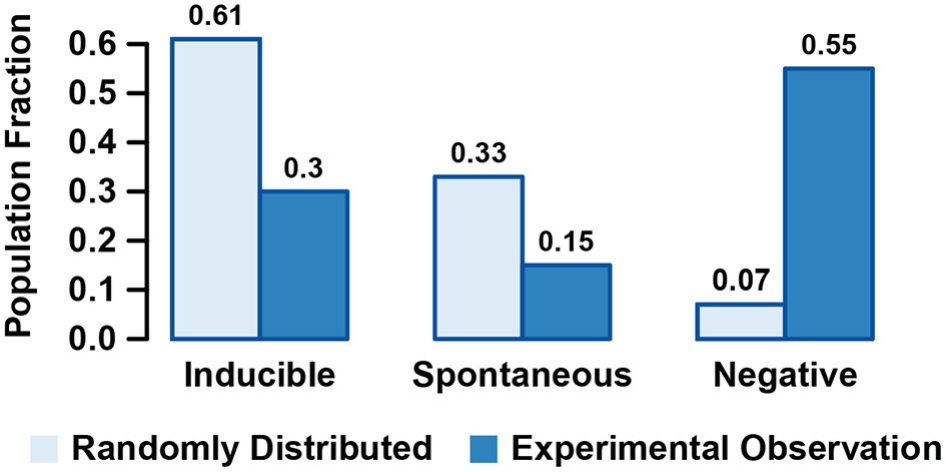
Fraction of states randomly selected based on an equiprobable random distribution that are contained in the basins of attraction of Inducible, Spontaneous, and Negative attractors and comparison with the experimental observed fraction of sperms displaying induced, Spontaneous, or Negative acrosome reaction (AR).

As shown by Figure 5, the proportion of initial states reaching Inducible, Spontaneous, and Negative attractors departed from the experimental observed proportions of cells undergoing Pg-induced AR, spontaneous AR, and no AR at all. This discrepancy indicates that it is unrealistic to assume a uniform equiprobable distribution of initial states, and that the set of initial states, interpreted as the heterogeneity in the initial sperm physiological states, must have a non-trivial distribution. Nevertheless, the results indicate that the physiological heterogeneity is closely related with the proportion of cells displaying AR, either spontaneous or induced.

To gain insight into the more realistic heterogeneity pattern, we selected the set of initial states by randomly drawing states from the basins of attraction of the Spontaneous, Inducible, and Negative attractors based on probabilities that matched the observed percentages of 15, 30, and 65%, respectively, and imposing the aforementioned restrictions on the fusion machinery nodes. Notice that the initial state selection procedure cancels any effect of the relative sizes of the basins of attractions and that by construction, the population of networks starting from this set of initial states will reproduce the observed proportion of spontaneous, inducible, and negative sub-populations. We used this set to complete our validation of the construction of the model as well as to investigate the putative common features and differences between those initial states. For that purpose, we explored first the activation probability P(x) of every node x in the network. That is:

- For Boolean nodes, we calculated the probability that a node x is in active state P(x) = P(x = 1) within the set of initial states.
- For the non Boolean nodes pH_i_, CatSper, [Ca^2+^]_i_ and Em, P(x) = P(x>M_x_/2), where M_x_ is the maximum value that the node x can take.

**Figure 6:**
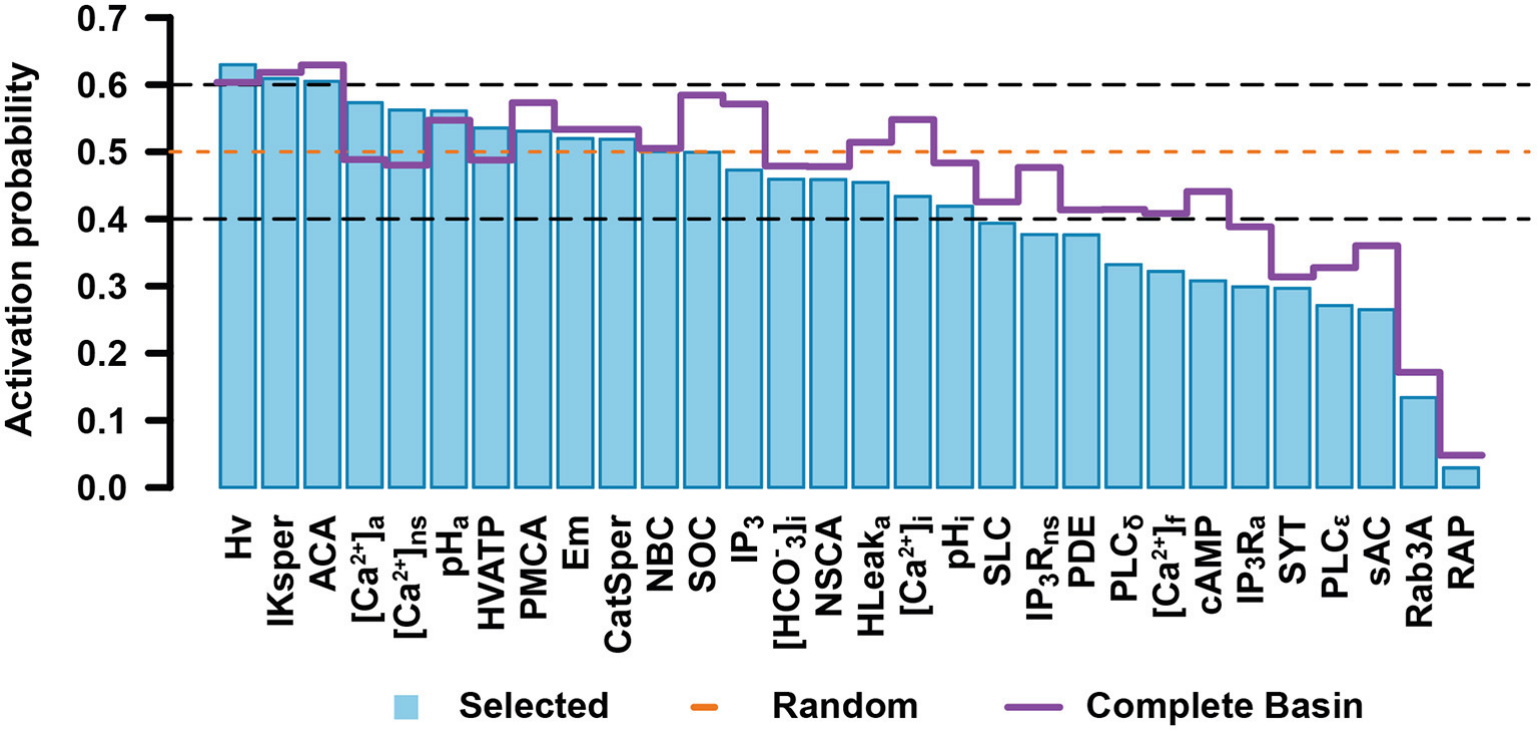
Activation probability of network nodes in the set of initial states. Bar heights for each node represent the probability that the node is in the active state. The orange dashed line points out the activation probability of a completely random set of initial states. The purple straight line represents the activation probability of each node on the entire set of states conforming the basins of attraction for all the attractors. Nodes whose activation probability is between 0.4 and 0.6 are enclosed in black dashed lines. Error bars are not shown since in each case standard deviation is below 0.001.

Figure 6 shows the activation probability of each node in the network (excluding nodes that represent external medium components). To have good statistics, we sampled 2 × 10^7^ different initial states constructed as described above, which kept the standard deviation of the activation probability below 0.001. The nodes can be separated into two main groups: those whose activation probability is between 0.4 and 0.6, not biased to activation or inactivation, and those that have a lesser than 0.4 or larger than 0.6 activation probability, therefore having a strong bias toward being either activated or inactivated. In agreement with the literature (Sosa et al., 2016), nodes downstream of the cAMP signaling cascade (RAP, Rab3A, cAMP, sAC) as well as IP_3_R Ca^2+^ channels were predominantly inactive. On the other hand, IKsper displayed a tendency to be active, which would be required to maintain the membrane hyperpolarized (Chávez et al., 2013). In addition, the acrosomal Ca^2+^ ATP-ase (ACA) and H^+^ channel Hv show high biases toward activation. ACA helps to maintain high concentrations of Ca^2+^ inside the acrosome, required for AR, while Hv increases pH_i_, an event related to CatSper activation and also likely necessary for AR (Darszon et al., 2006; Lishko et al., 2012; Miller et al., 2018). Figure 6 also shows the activation probabilities of the nodes in the selected set of initial states (blue bars), in an equiprobable random set of initial states (dashed orange line) and in all the states composing the basins of attractions of all the attractors of the system (purple line). The node activation pattern of the selected initial states diverges from the random initial states pattern in most of the nodes, especially on those nodes downstream of the cAMP signaling cascade, nodes related to Ca^2+^ mobilization as IP_3_R_a_, PLC, ACA, and nodes linked to pH_i_ and Em regulation as Hv and IKsper, respectively. The difference between the node activation patterns of the selected initial states and the states in the entire basins of attraction is also noticeable; mainly, the nodes related to cAMP and [Ca^2+^]_a_ signaling cascades show lower activation in the selected set of initial states. This is likely because in the Spontaneous and Inducible attractors, the activation of these nodes favors the activation of the reporter node Fusion. Consequently, the states where cAMP and [Ca^2+^]_a_ regulation-related nodes are active are congregated around the attractor. This is the reason that the entire set of states in the basin of attraction considers more states with activation of these nodes than the selected set of initial states, explaining activation probability pattern differences.

**Figure 7:**
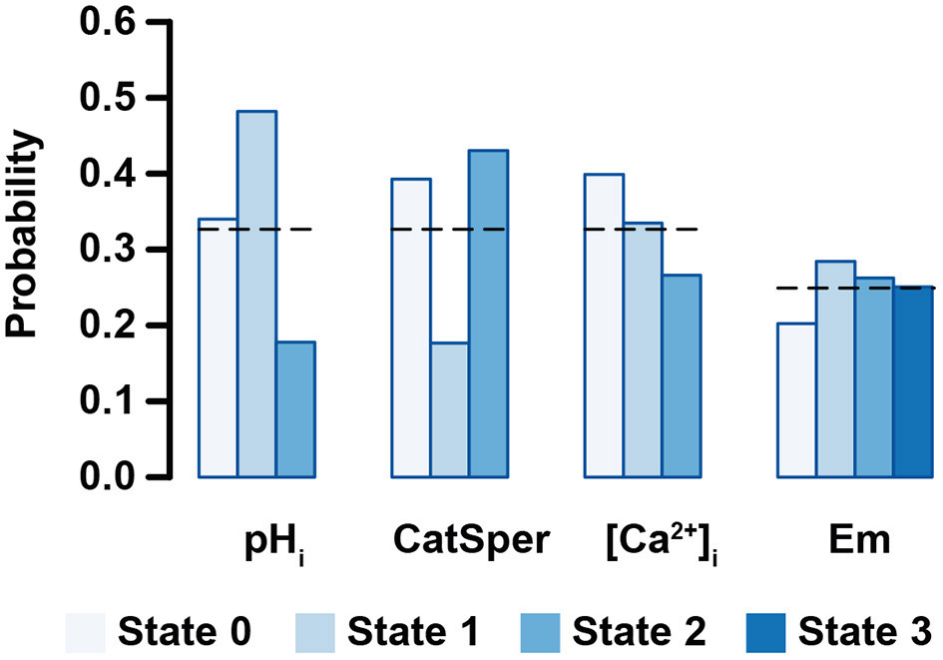
Probability that one node takes a specific value in the set of initial states for non-binary nodes. Bar lengths show the average probability calculated over 2 × 10^7^ initial states. Black dashed lines indicate the probability on a completely random set of initial states. Biological interpretation for each node is as follows: pH_i_ (acidic 0, mildly alkaline 1, fully alkaline 2); CatSper (closed 0, open 1, inactivated 2); [Ca^2+^]_i_ (basal 0, activator 1, inhibitor 2); Em (hyperpolarized 0, equilibrium 1, mildly depolarized 2, and fully depolarized 3). Error bars are not shown since standard deviation is below 0.001.

**Figure 8:**
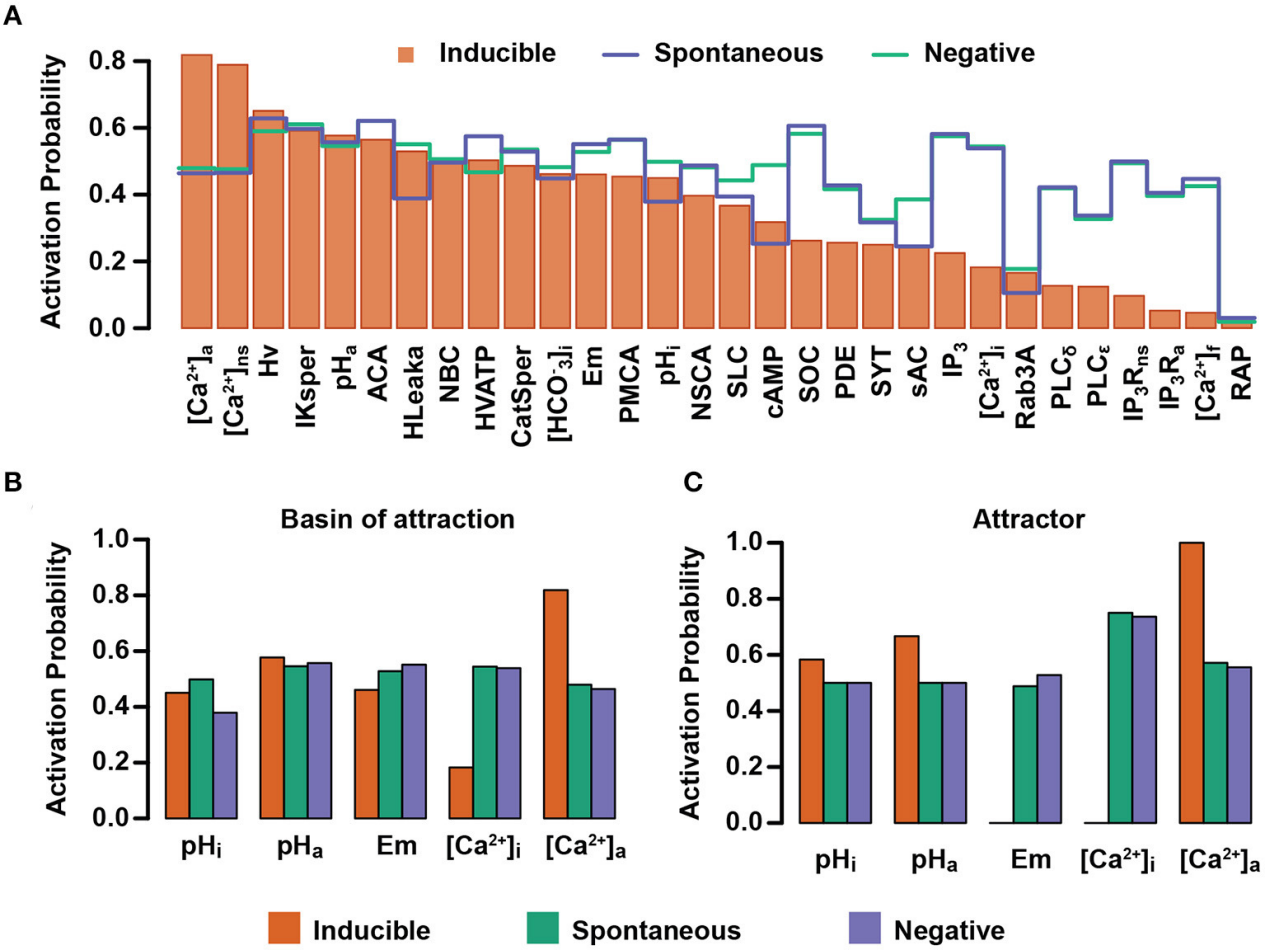
Activation probability of network nodes in Inducible, Spontaneous, and Negative attractors. **(A)** Bars represent the activation probability of the network nodes for states in the Inducible attractors. Blue and green lines correspond to the activation probability of the network nodes in the Spontaneous and Negative attractors, respectively. **(B,C)** Activation probability of pH_i_, pH_a_, Em. [Ca^2+^]_i_ and [Ca^2+^]_a_ in the basins of attraction **(B)** or attractors **(C)** in the three functional classes.

For non-binary nodes, Figure 7 shows the probability that a node takes a specific value in the set of initial states. As can be seen, only a small population of initial states take the most alkaline pH_i_, while most initial states take more acidic values. This agrees with the notion that cytosolic alkalinization is required for AR (Florman et al., 1989; Darszon et al., 2006; Lishko et al., 2012). Most of the initial states in the population belong to the Negative attractor group that will not lead to either spontaneous or induced AR. Also, only small fractions of the set of initial states show the open state (1) for CatSper and the high [Ca^2+^]_i_ levels, agreeing with the idea that CatSper activation is important to increase [Ca^2+^]_i_ and AR (Baldi et al., 2009; Miller et al., 2016). No significant differences can be appreciated among the Em values, although this could be compensated by the activity of IKsper shown in Figure 6 to display a strong tendency of Em toward hyperpolarized values in the Inducible attractors (Figure 8). These results suggest that the proportion of a sperm population displaying spontaneous, Pg induced AR or no AR, is related to the sperm physiological state heterogeneity. Furthermore, this heterogeneity is not arbitrary and there are cell elements more robust to perturbations than others.

As mentioned above, to further validate the model and to better characterize the selected set of initial states, we compared the results of the model with a set of 26 different experimental observations reported in the literature and described in Table 2. We focused our attention on the behavior of cAMP, pH_a_, pH_i_, Em, [Ca^2+^]_i_, [Ca^2+^]_a_, and the reporter node Fusion, given their relevance for the AR. For each variable, we selected a set of experimental observations reported on the literature and replicated the experiments in the model. Recall that in our approach, each sperm is running its own internal network in a particular initial state. To simulate a sperm population incubated under a specific experimental condition (EC), we used a population of networks, each starting in one of the aforementioned initial states, replicated the effects of the EC and let the dynamics run until each network reached its corresponding attractor. We measured the average effect of each EC and qualitatively compared the behavior of the network population with the reported experiment. Appendix B of the Supplementary Material shows two examples of how the simulations and comparisons were constructed. Table 2 shows that our model agrees with 92% of the experimental results obtained by different research groups.

**Table 2:** Experimental observations compared with the behavior of the acrosome reaction (AR) discrete network.

**Variable**	**Condition**	**Reference**	**Reported**	**Model**		**Variable**	**Condition**	**Reference**	**Reported**	**Model**
**cAMP**						**[Ca**^**2+**^**]**_**a**_				
	Capacitation	Visconti et al., 1995, 2002	↑	↑ 2%			Pg	Rossato et al., 2001; De Blas et al., 2005	↓	↓ 9%
	Pg	Sosa et al., 2016	↑	↑ 3%		**Swelling**				
**pH**_**a**_							Control	Sosa et al., 2016	↑ 15%	↑ 30%
	Capacitation	Nakanishi et al., 2001	↑	↑ 3%			XC + A23187	Sosa et al., 2016	↑	↑ 61%
	DCCD	Nakanishi et al., 2001	↑	↑ 45%			XC + TG	Sosa et al., 2016	↑	↑ 61%
	DIDS	Nakanishi et al., 2001	↓	↓ -1%			XC + cAMP	Sosa et al., 2016	↑	↑ 70%
	NNC	Chávez et al., 2017	↑	↑ 45%			XC + 8-pCPT	Sosa et al., 2016	↑	↑ 70%
	NNC vs Ionomycin	Chávez et al., 2017	↑	↑ 50%			XC + Rab3A	Sosa et al., 2016	↑	↑ 70%
**pH**_**i**_							XC + Pg	Sosa et al., 2016	↑	↓ -3%
	Capacitation	Darszon et al., 2006; Lishko et al., 2011	↑	↑ 5%		**AR**				
**Em**							DCCD	Nakanishi et al., 2001	↑	↑ 55%
	Capacitation	Chávez et al., 2013	↓	↓ -86%			DIDS	Nakanishi et al., 2001	↑	↑ 7%
**[Ca**^**2+**^**]**_**i**_							Ionomycin	Chávez et al., 2017	↑	↑ 28%
	Capacitation	Lishko et al., 2011; Visconti et al., 2002	↑	↑ 5%			NNC	Chávez et al., 2017	↑	↑ 85%
	Pg	Blackmore et al., 1990; Kirkman-Brown et al., 2000	↑	↑ 24%			NNC vs Ionomycin	Chávez et al., 2017	↑	↑ 57%
	NNC	Chávez et al., 2017	↑	↑ 28%						
	Ionomycin vs NNC	Chávez et al., 2017	↑	↑ 20%						

*For each variable, we replicated in the model a series of experimental conditions reported in the literature. Arrows indicate the qualitative change in the measured variable (↑ increase, ↓ decrease) when subjected to each condition. Agreement and disagreement between the experimental observations and the network behavior are highlighted as green rows, indicating a similar qualitative response in the experiments and the model, and red rows where dissent was found. Percentages indicate the magnitude of the change related to the maximum possible response. The particular case of Swelling under the Control condition is highlighted as a disagreement since the percentage of swollen acrosomes in the model is significantly higher than reported. All the experiments were performed in different capacitating mediums (see references). DCCD, N,N′-dicyclohexylcarbodiimide; DIDS, 4,4′-diisothiocyanato-stilbene-2,2′-dislufonic acid; NNC, NNC55-0396; XC, Xestospongin C; TG, thapsigargin; 8-pCPT, 8-pCPT-20-O-Me-cAMP*.

### 3.3. Spontaneous, Inducible and Negative Attractors Show Large-Scale Differences on the Network Physiological State

Once having validated our network model, we proceeded to examine its prediction capacity. To this end, we further analyzed the characteristics that define the functional classification of the attractors into the Spontaneous, Inducible, and Negative AR. We calculated the activation probability of all the nodes for each functional group within the set of initial states previously constructed. Figure 8A shows the nodes where the activation probability is significantly different among the three functional groups. The biggest differences are shown in the Inducible class, where activity of nodes related to [Ca^2+^]_i_ and [Ca^2+^]_a_ have the most significant change in the activation probability compared with the other two classes. Although differences between Negative and Spontaneous attractors are subtle, it can be seen that there are considerable changes in the activation probability for sAC, the acrosome H^+^ outward current (HLeak), pH_i_, the acrosomal V-ATPase, and Em. Notably, this figure shows that, in the set of initial conditions, the nodes related to pH_i_ and pH_a_ regulation display different activation probabilities among the functional classes.

We focused on the pH_i_, pH_a_, Em, [Ca^2+^]_i_ and [Ca^2+^]_a_ nodes. Figure 8B shows there are significant differences in the activation probability of these nodes between functional classes. As expected, the pH_i_ node displays a higher activation probability in the Spontaneous than in the Inducible attractors, being the least likely in the Negative class. No significant differences can be appreciated regarding the pH_a_ node activation probability among the three classes. Remarkably, the Em and [Ca^2+^]_i_ nodes reach their lowest activation probabilities in the Inducible class, indicating that increases in Em activity (depolarization) and in [Ca^2+^]_i_ activity (increased concentration) often lead the system to attractors that display spontaneous fusion activity or no fusion activity at all. The [Ca^2+^]_a_ node shows its highest value also in the Inducible class, indicating that low [Ca^2+^]_a_ node activity drives the network to the Spontaneous or Negative attractor. This behavior is mostly preserved when we apply the same procedure, not to the entire basin of attraction, but to the attractor itself, calculating the activation probability of the nodes within the attractor's states. As Figure 8C shows, differences in this case are more evident between classes; the pH_i_ and pH_a_ nodes display their highest activity levels in the Inducible attractor. We found that in all the network states of the attractors in this class, Em is at its lowest value (Em = 0), representing hyperpolarization, which means that whenever an attractor state contains a value for Em different than 0, the dynamics drive the network either to the Spontaneous or Negative attractor. Similarly, the [Ca^2+^]_i_ node always takes the basal value 0 in attractors of the Inducible class. This does not oppose established evidence that [Ca^2+^]_i_ rises during capacitation but indicates it must remain at basal levels while awaiting for a stimulus such as Pg to trigger AR; otherwise, an increase in the activity of the [Ca^2+^]_i_ node drives the network to a Spontaneous or Negative attractor. As expected, the [Ca^2+^]_a_ node activity tends to be lower within the Spontaneous attractor and it reaches its highest value in the Inducible attractor.

These results are consistent with the notion that AR reaction is promoted by increases in [Ca^2+^]_i_ as well as membrane depolarization, but they also suggest the existence of hyperpolarized sperm with elevated [Ca^2+^]_i_ that do not react. The activation probability of the [Ca^2+^]_a_ node in the Inducible class is consistent with the idea that acrosomal stability requires high Ca^2+^ levels to prepare sperm for the Pg stimulus, and contributing to maintain low [Ca^2+^]_i_. Also, the pH_i_ node activation probability pattern displayed in the functional classes agrees with the notion that a cytosol alkalinization enables spontaneous as well as Pg-induced AR, but the model predicts a more elevated pH_i_ in cells that can respond to Pg than in cells that undergo AR spontaneously.

### 3.4. Elevation of pH_a_ Defines Network Capacity for AR

**Figure 9:**
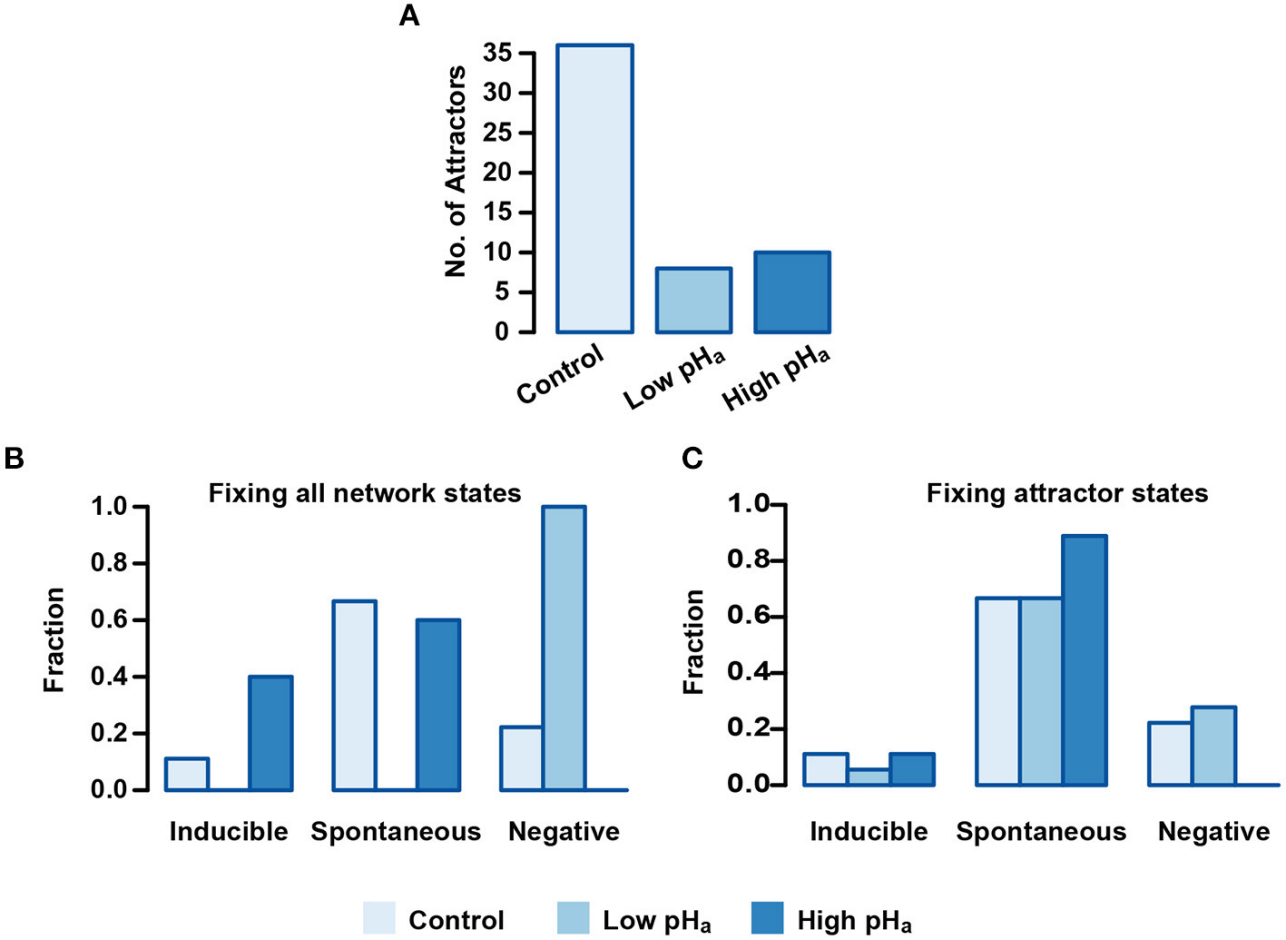
pH_*a*_ blocking effect on Inducible, Spontaneous, and Negative basins of attraction. **(A)** Number of total attractors when the pH_*a*_ node is fixed as acidic (0) or alkaline (1) on the entire state space. **(B)** Distribution of attractors among functional classes fixing pH_*a*_ as in **(A)**. Setting pH_*a*_ = 0 results in the deletion of Inducible and Spontaneous attractors, whereas fixing pH_*a*_ = 1 leads to deletion of the Negative class. **(C)** Attractors distributed among the different functional classes where pH_*a*_ is fixed only in the attractor states. Fixing pH_*a*_ = 1 on the sequence of Negative attractors transforms them in Spontaneous attractors.

To further investigate the role of pH_a_ during acrosomal exocytosis, we analyzed how the attractor landscape changes as a consequence of acidic (pH_a_=5.5) or alkaline (pH_a_=6.7) conditions by fixing the pH_a_ node value to 0 or 1, respectively. We noticed first that fixing the pH_a_ node drastically reduced the number of total attractors, as shown in Figure 9A. Without determining a particular value for pH_a_, and setting Pg=0, which in our model represents absence of Pg, the dynamics generate a total of 36 attractors that are reduced to 8 and 10 in conditions representing low (0-acidic) and high (1-alkaline) pH_a_, respectively. Remarkably, the extent of this contraction does not follow from the fact that fixing the value of pH_a_ reduces by half the total number of possible network states. This change implies that fixing pH_a_ limits the attractor landscape and therefore reduces the possible different stable behaviors that the network can reach from any of the available initial states.

To better understand the repercussions of this landscape contraction, we re-classified the resulting attractor landscape according to the functional classes and we calculated the fraction of the total attractors that resides in each one. Figure 9B shows that for pH_*a*_ = 0, no attractor displays activation of the reporter node Fusion on any of its states, and the entire attractor landscape falls into the Negative AR class. On the other hand, the Negative class vanishes when pH_*a*_ = 1 and all attractors in the landscape display activation of the reporter node Fusion, either in the Spontaneous or Inducible class. Noticeably, only Inducible attractors increase with respect to the control group in this condition, suggesting that only the induced AR is promoted by acrosomal alkalinization.

Constraining the pH_*a*_ node to a fixed value on the entire state space is a major perturbation of the system since it trims the space of available states and reduces the total number of attractors. Considering this, we also analyzed a less drastic perturbation: instead of fixing pH_*a*_ on all possible states, we fixed it only on the states conforming to the original 36 attractors. This would mean altering pH_*a*_ once the cell reached an attractor after being capacitated. As shown in Figure 9C, this perturbation has less drastic effects on the functional distribution of the attractors, but still, when pH_*a*_ = 0, the number of Inducible attractors decreases and the number of Negative attractors increases, while the number of Spontaneous attractors remains the same. This means that the dynamics under this condition reduces the capacity of the network to activate the reporter node Fusion under Pg activation. In contrast, the Negative class disappears when pH_*a*_ = 1 and the number of Spontaneous attractors increases while the Inducible class remains intact. These findings indicate that a pH_*a*_ increase can by itself trigger AR in a group of cells, but also, that it increases the number of cells that make AR induced by Pg.

### 3.5. Calcium and pH Transitory Perturbations Promote Functional Changes With the Highest Probability

At this point, we should recall that the dynamics in our model, described by equation (1) is deterministic and synchronously updated. This means that each initial state leads to one and only one attractor. As a further step in understanding how robust the network is to small transitory changes in pH_*a*_, as well as how it compares to the effect of the same kind of changes in other nodes, we implemented a series of small node-specific perturbations and we counted the fraction of those perturbations that promoted not only a change in the residing basin of attraction but also in the functional class of the resulting attractor. To this purpose, we took a set of 10^5^ random states in the basin of attraction of each attractor and changed the value of one single node, the same for all states. In the case of binary nodes, we simply inverted the value for its complement; for non-binary nodes, we randomly took a different value among the set of possible node values. In the particular case of the Spontaneous class, given the irreversibility of membrane fusion, we took our sample only from those states that did not translate into a final AR stage, that is, we only selected those conditions where the nodes Swelling, SNARE's, SYT and the reporter node Fusion had a value of 0.

**Figure 10:**
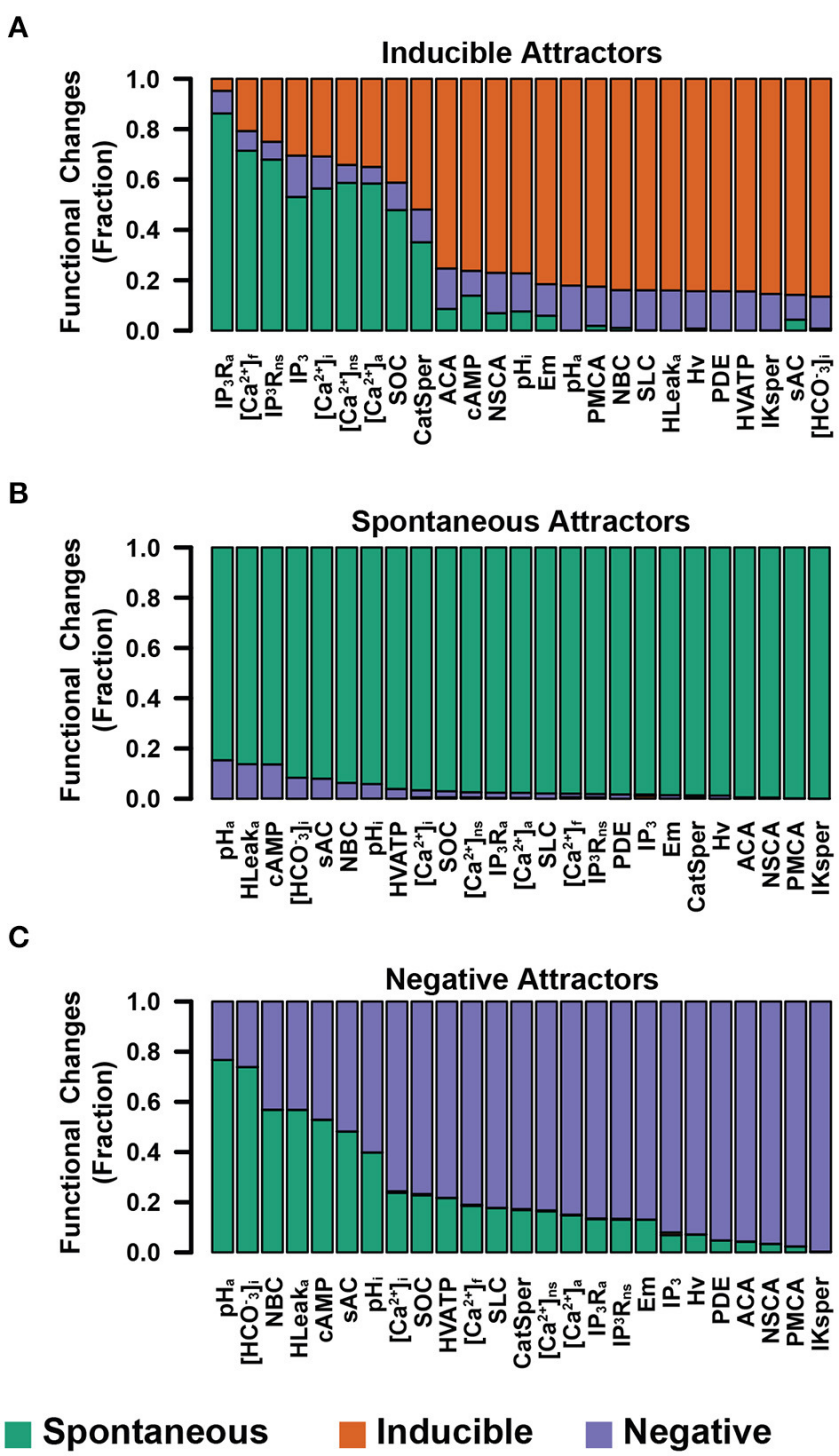
Fraction of node-specific perturbations that promoted functional changes on Inducible **(A)**, Spontaneous **(B)**, and Negative **(C)** attractors. Nodes involved directly in [Ca^2+^]_*i*_ and [Ca^2+^]_*a*_ regulation show high sensitivity on the Inducible group, whereas Spontaneous and Negative attractors show higher sensitivity to perturbations to nodes related to pH_*a*_ and pH_*i*_. The three panels show a minimal change into Inducible attractors from Negative and Spontaneous groups.

The results shown in Figure 10 present in order the nodes where the perturbations had the highest rates of change in the attractor type for the different functional classes. As shown in panel A, the nodes that made the Inducible attractors more susceptible to perturbation were those related to the regulation of the [Ca^2+^]_*i*_ and [Ca^2+^]_*a*_. Remarkably, pH_*a*_ node perturbations did not promote a significant number of functional changes. Also, most of the changes promoted were to the Spontaneous class. Panels B and C show that in the cases of Spontaneous and Negative attractors, the nodes most susceptible to perturbation were mainly pH_*a*_ and those related to pH_*i*_ and pH_*a*_ regulation. Panel B illustrates that there is only a small amount of changes produced by perturbations in the Spontaneous attractors that can lead the network to the Negative class. Noticeably, most of the changes in the Negative attractors were directed toward the Spontaneous attractors as shown in panel C. This result predicts that changes in pH_*a*_ can promote AR, at least partially, without any other stimuli and that most of the sperm reacted in this way come from a group of sperm that would have not displayed Spontaneous or Pg-induced AR. The results presented in this section indicate that not only sustained changes promote AR but also transitory stimuli, mostly in Ca^2+^ and pH in cytosol and acrosome, are important as inducers of the AR. We address this topic further in the discussion.

## 4. Discussion

In this work, we developed a discrete, synchronous, and deterministic regulatory network for the acrosome reaction in human sperm. The knowledge on the physiological processes that culminate in the fusion of the outer acrosomal membrane and the plasma membrane is incomplete, and some of the evidence is controversial. Despite the dearth of knowledge, the network presented here considered the most recent literature on the main intracellular components and interactions involved in the acrosome reaction, hoping to promote a better understanding of this process.

From the calculation of the attractor landscape and the identification of the states that belong to their corresponding *basins of attraction*, we concluded that this network has only cyclic attractors representing stable physiological oscillations; there are no fixed points. We noticed that these attractors are modified by the switching on the node representing extracellular Pg, and that these differences are consistent with the Pg response of a sperm population. Taking into account this finding, we partitioned the attractor landscape in Spontaneous, Inducible, and Negative attractors based on their functional characteristics.

As is common in the logical dynamics literature (Mendoza and Alvarez-Buylla, 2000; Espinosa-Soto et al., 2004; Álvarez-Buylla et al., 2008; Espinal et al., 2011; Espinal-Enríquez et al., 2017), we addressed the probabilities of reaching different attractors by taking random and uniformly distributed initial states from the state transitions graph. This approach led us to conclude that the proportions of network populations reaching spontaneous and Pg-induced AR are inconsistent with the proportions observed experimentally (Nakanishi et al., 2001; Stival et al., 2016). We explored the characteristics of different sets of initial states that were more consistent with proportions of AR experimentally reported under physiological conditions. We determined that the activation probability of each node in this set of initial states agrees with the biological behavior reported in the literature. This suggested that not all of the possible network states are reachable under physiological conditions. Moreover, the number of attractors displayed by the network and the size of their basins of attraction is not physiologically relevant. With our study, we were able to delimit the set of network states that should be considered as potential states characteristic of capacitated sperm.

Furthermore, our results showed that, within the set of initial states, a group of nodes displayed an activation probability close to 0.5, meaning that it is equally probable that they take any of their possible values, while for other nodes the activation probability has a strong bias toward either basal levels (0) or increased activity (>0). In all the sets of initial states analyzed, these activation patterns are required in order to reproduce the experimental proportions of spontaneous AR, Pg-induced AR, and no AR at all. This suggests that there is a relation between the structure and characteristics of the heterogeneity in the physiological states in a sperm population and how this population is partitioned to display the observed proportions. As stated before, our model considers events between later stages of capacitation and membrane fusion and it is not suitable to investigate the origin of population heterogeneity, how it changes during the different stages in sperm development and maturation and the order of events that finally promote these activity patterns. To address these matters, our model can be expanded to consider the events that take place during early capacitation stages or even spermatogenesis. This would allow us to evaluate the properties of heterogeneity at different moments during the sperm maturation processes.

We showed that the model can qualitatively reproduce experimental observations reported in the literature. In this sense, our model has an overall success rate of 92% in reproducing 24 of 26 experimental results describing different behaviors of a sperm population in response to diverse conditions and stimuli.

An experimental observation our model was unable to reproduce is the finding that Pg increases acrosomal swelling in a population of human sperm in the presence of xestopongine C (XC), an IP_3_R blocker (Sosa et al., 2016). In our model, Pg promotes only the activity of CatSper in the sperm flagellum (Meizel et al., 1997; Baldi et al., 2009). We hypothesized that the local increase in flagellar [Ca^2+^]_*i*_ and its diffusion to the head is insufficient to promote the activation of the signaling cascade that triggers the fusion machinery, which involves a Ca^2+^-dependent cAMP increase and a Ca^2+^-dependent IP_3_R channel. Instead, we envisage that the local Ca^2+^ wave generated in the flagellum is re-transmitted by IP_3_R located in the neck stores and that this re-transmission, being closer to the head, provides the necessary Ca^2+^ to activate AR. However, in presence of XC this would not be possible, as this inhibitor would stop re-transmission from the neck stores, and thus the flagellar Ca^2+^ signal would not reach the head, preventing acrosome swelling and AR to occur. This result could suggest the participation of a different channel involved in the retransmition of the Ca^2+^ signal, located in the neck stores. For instance, evidence indicates that the Ryanodine receptors (RyRs), located in the redundant nuclear envelope in human sperm, are involved in the generation of intracellular spontaneous and Pg-induced [Ca^2+^]_*i*_ oscillations (Mata-Martínez et al., 2018). However, the role and expression of RyRs in human sperm functioning has been controversial (Harper et al., 2005; Bedu-Addo et al., 2008; Teves et al., 2009; Mata-Martínez et al., 2018). The discrepancy between our model and this particular experiment could also suggest that Pg promotes a [Ca^2+^]_*i*_ increase in the head or activates AR by a different pathway. It has been shown that Pg promotes [Ca^2+^]_*i*_ increases through CatSper in humans (Lishko et al., 2010; Strünker et al., 2011), nevertheless this hormone does not activate CatSper in mice (Mannowetz et al., 2017; Orta et al., 2018), although it can still promote AR (Murase and Roldan, 1996; Nahed et al., 2016). However, the alternate signaling path triggered by Pg in the head and its relation to the AR is unknown.

Next, we calculated the probability that each node in the network is active in the Spontaneous, Inducible, and Negative attractors. The differences in the activation probability give us insight on the differences in the physiological state of sperm and their capacity to reach AR. We found that the attractors in the Inducible group have notable differences, especially in the Em, [Ca^2+^]_*i*_, [Ca^2+^]_*a*_, pH_*a*_ and pH_*i*_ nodes, which is consistent with the literature.

We tested the behavior of the attractor landscape distribution among functional classes to identify how attractors are affected by changes in the state of the pH_*a*_ node. We found that the total number of attractors is drastically reduced when low and high pH_*a*_ conditions are simulated and applied to the entire space of possible network states. As this is a drastic maneuver, we also tested fixing the value of the pH_*a*_ node only in the states that conform the attractor to determine how attractors distributed among the functional classes, while preserving the total number of attractors. In both cases, we found that the conditions where pH_*a*_ = 0 increase the number of Negative attractors, preventing the network from activating the reporter node Fusion. This means that acidic pH_*a*_ conditions would partially prevent AR. Remarkably, when pH_*a*_ = 1, the Negative attractors disappeared and were transformed into Inducible or Spontaneous attractors. These results suggest that for a fraction of the sperm population, acrosomal alkalinization acts as a necessary step for AR induction. In addition, they indicate that a pH_*a*_ rise is partially promoting AR by itself in a group of sperm. This model prediction is supported by published work from our group (Chávez et al., 2017).

We then applied a series of small and transitory perturbations to the states in the basins of attraction of the different functional classes of attractors to identify the network elements that are more sensitive to perturbations. We explored if the temporary alteration of a single node could change the trajectory of the network toward an attractor of a different functional class. As it is well known that AR is effectively induced by increasing [Ca^2+^]_*i*_ in capacitated sperm (Baldi et al., 2009; Miller et al., 2016), we found that nodes related to [Ca^2+^]_*i*_ and [Ca^2+^]_*a*_ regulation show the highest sensitivity to perturbations and most changes in these nodes promote AR. These findings are consistent with experimental results. Noticeably, we found that pH_*a*_ is the most sensitive node for the Spontaneous and Negative attractors, followed by nodes related to pH_*i*_ and pH_*a*_ regulation.

We made a preliminary evaluation of the effect of small perturbations to the regulatory functions by first considering the set of initial states that display physiological proportions of Spontaneous and Inducible AR (15 and 30%, respectively). Then, one at a time, we applied perturbations to the regulatory logic of different nodes by changing the outcome of one single row of the respective truth table (see Appendix A of the Supplementary Material). We recalculated the proportions of Spontaneous and Inducible AR under the same set of initial states. Preliminary results show that most of these perturbations have little or no effect on the original AR proportions. Only those nodes with few regulators close to the Fusion reporter node (e.g., Rab3A) are sensitive to small changes in their regulatory logic. However, examining the sensibility of the model would require a detailed analysis of all nodes, an endeavor worth pursuing in future work.

Novel information and data can be integrated into the model, helping to clarify the signaling cascades participating in the human sperm acrosome reaction. For instance, actin polymerization dynamics play a vital role in the AR. During capacitation, G-actin polymerizes on the sperm's head, preventing untimely AR. Later, actin filaments depolymerize, allowing acrosome swelling and membrane fusion (Liu et al., 1999; Breitbart et al., 2005; Romarowski et al., 2018). We considered these dynamics in the swelling node that require [Ca^2+^]_*i*_ and cAMP increases to activate. However, insufficient knowledge on the complex actin patterns and dynamics that influence AR (Romarowski et al., 2016) precluded incorporating this important process in our present model, but will be a subject in future work.

Synchronous and deterministic discrete updating are limitations of this model. Although many sperm elements update their state synchronously (e.g., Em reacts instantly to ionic current changes), this is not the case for others. Quantitative fine-tuning to AR data require more complex models involving continuous variables, stochasticity, and noise. For example, Pg entails a biphasic [Ca^2+^]_*i*_ elevation necessary for AR (Kirkman-Brown et al., 2000; Sánchez-Cárdenas et al., 2014), while [Ca^2+^]_*i*_ oscillations seem to inhibit it (Sánchez-Cárdenas et al., 2014; Mata-Martínez et al., 2018; Torrezan-Nitao et al., 2021). Simons et al. proposed a differential equation model to reproduce these [Ca^2+^]_*i*_ patterns (Simons and Fauci, 2018). Modeling actin dynamics would also require spatiotemporal-dependent variables.

Furthermore, discrete models can be extended to become more complex and descriptive, for instance to study [Ca^2+^]_*i*_ spikes in sea urchin sperm (Priego-Espinosa et al., 2020). In this way, our model is a stepping stone to deal with the complexity of the physiological AR processes quantitatively.

Overall, our model captures the essential aspects of the interactions involved in the AR rather than focusing on their kinetic details, reproduces experimental observations, and allows us to study the fundamental properties of this complex process. Our results show that the characteristics of the physiological heterogeneity in a sperm population directly influence the proportion of sperm capable of undergoing the AR, either spontaneous or stimulated by Pg. Also, our findings suggest that not only long-term pH_*a*_ changes induce AR, also transient perturbations promote this reaction in a fraction of the sperm population.

## Data Availability Statement

The original contributions presented in the study are included in the article/Supplementary Material, further inquiries can be directed to the corresponding author/s.

## Author Contributions

AA, JC, GM-M, and AD conceptualization, investigation, methodology, formal analysis, software development, writing review, and editing. All authors contributed to the article and approved the submitted version.

## Conflict of Interest

The authors declare that the research was conducted in the absence of any commercial or financial relationships that could be construed as a potential conflict of interest.
