# Supplementary material for: Discrete Dynamic Model of the Mammalian Sperm Acrosome Reaction: The Influence of Acrosomal pH and Physiological Heterogeneity
Source: Front Physiol. 2021 Jul 19;12:682790. doi: 10.3389/fphys.2021.682790 (PMC8328089; doi:10.3389/fphys.2021.682790)
Supplement: Supplementary file 1 [file Data_Sheet_1.pdf]

Supplementary Material

Appendix A: Example of the construction of IP_3R_a regulatory function as an example

Acrosomal IP_3 receptors (IP_3R_a) are $[Ca^{2+}]_i$ and IP_3 dependent Ca^{2+} channels located in the outer acrosomal membrane. They possess one IP_3 binding site and two Ca^{2+} binding sites of high and low affinities that open or block the channel respectively. Increasing $[Ca^{2+}]_i$ in presence of IP_3 promotes channel opening. A further increase in $[Ca^{2+}]_i$ blocks the channel by means of the low affinity Ca^{2+} binding site.

Table S1 presents the regulatory function of the IP_3R_a. For its construction, we first identified the nodes IP_3 and $[Ca^{2+}]_i$ as its regulators. IP_3 can take only two values (Basal=0 and Increased=1) while $[Ca^{2+}]_i$ can take three values (Basal=0, Activator=1, Inhibitor=2). $[Ca^{2+}]_i$=1 represents an increase in $[Ca^{2+}]_i$ sufficient to open the IP_3R_a in presence of an IP_3 increment, while $[Ca^{2+}]_i$=2 promotes its blockade. For practical purposes, the IP_3R_a can take one of two possible values (Open=1 and Closed=0), depending on the value of its regulators. When IP_3=0, the channel can not open independently of the value of $[Ca^{2+}]_i$. When $[Ca^{2+}]_i$=0 or $[Ca^{2+}]_i$=2, the channel is closed because $[Ca^{2+}]_i$ is either too low or too high, independently of the value of IP_3. This means that IP_3R_a=1 only when IP_3=1 and $[Ca^{2+}]_i$=1. The value of IP_3R_a and in general of every node should be specified for every possible value of its regulators.

**Regulators**		**Target**
**IP_3**	**$[Ca^{2+}]_i$**	**IP_3R_a**
0	0	0
0	1	0
0	2	0
1	0	0
1	1	1
1	2	0

Table S1: IP_3R_a regulatory function. Rows represent the value assigned to IP_3R_a under each possible value of its regulators IP_3 and $[Ca^{2+}]_i$

Apendix B: Two examples of comparison between experimental observations and model results

Example 1: percentage of swollen acrosomes

In Sosa et al., 2016, the authors incubate human sperm samples in Xestospongin C (XC) with different treatments. In the presence of ionophore A23187 and Pg, the percentage of swollen acrosomes increases with respect to control. We reproduced the same experimental conditions in our model and measured the percentage of swollen acrosomes simulating the addition of XC, A23187 and Pg. For A23187, the difference of swollen acrosomes (61% normalized by the total population) is in agreement with the experimental result, which is reported in Table 2 as a green row and two arrows pointing in the same direction, indicating the same qualitative response between the model and the experiments (↑↑). In the case of Pg, the difference of swollen acrosomes (-3%) differ with the experimental observation, which is indicated as a red row and arrows pointing in opposite directions (↑↓) (Figure S1). We addressed the reasons of the disagreement in the discussion.

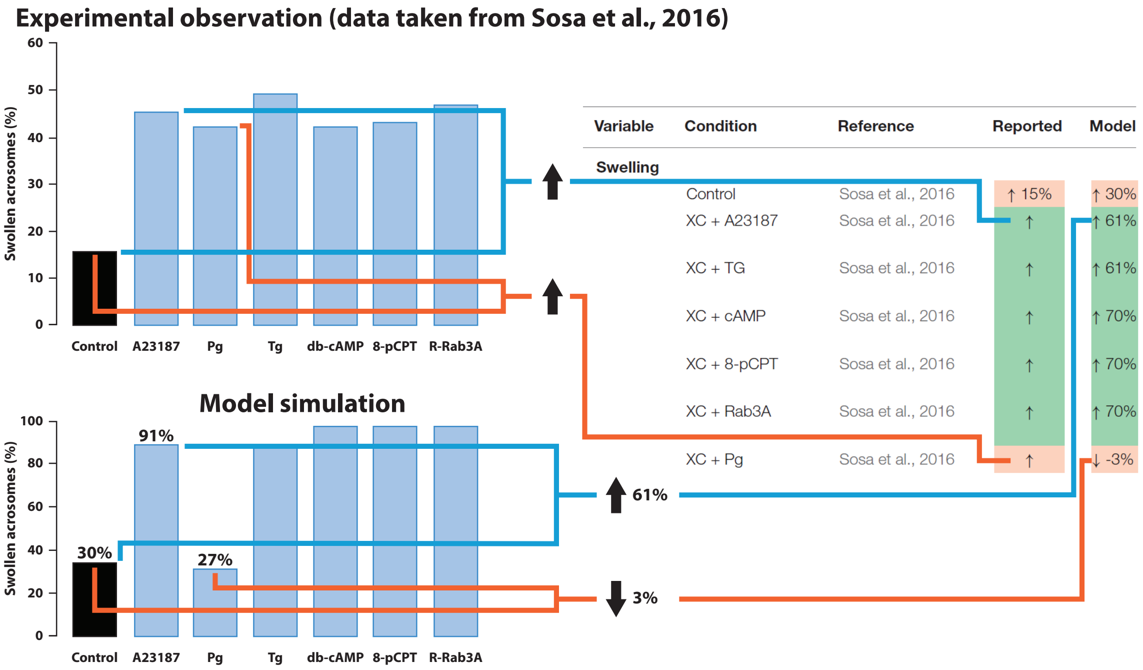

Figure S1: Comparison between model and experimental observations for swollen acrosomes in presence of XC, A23187 and Pg. Arrows indicate the sign of the difference in swollen acrosomes between A23187 or Pg, and Control conditions for reported experiments and model results. Agreement between the experiments and the model is indicated by a green row highlighting arrows pointing in the same direction, whereas disagreement is specified as a red row and arrows pointing in opposite directions. The magnitude of the change in the model is indicated as the percentage of the maximum response.

Example 2: $[Ca^{2+}]_i$ response to Pg

In Kirkman et al., 2000, the authors report a rise in $[Ca^{2+}]_i$ as a response to Pg stimulation. We simulated the experimental conditions in the model and measured the effect of Pg addition in the average normalized value of $[Ca^{2+}]_i$ taken in a sperm population, considering the last 20 time steps of the time series before and after Pg addition. Pg induced an increase in $[Ca^{2+}]_i$ in the model and the experiment, which we reported in Table 2 as a green row and two arrows pointing in the same direction, indicating the same qualitative response between the model and the experiments. The model was unable to reproduce the first $[Ca^{2+}]_i$ transitory increase, given the model limitations. Quantitative reproduction of the observed experiments require further complexity into the model, as addressed in the discussion.

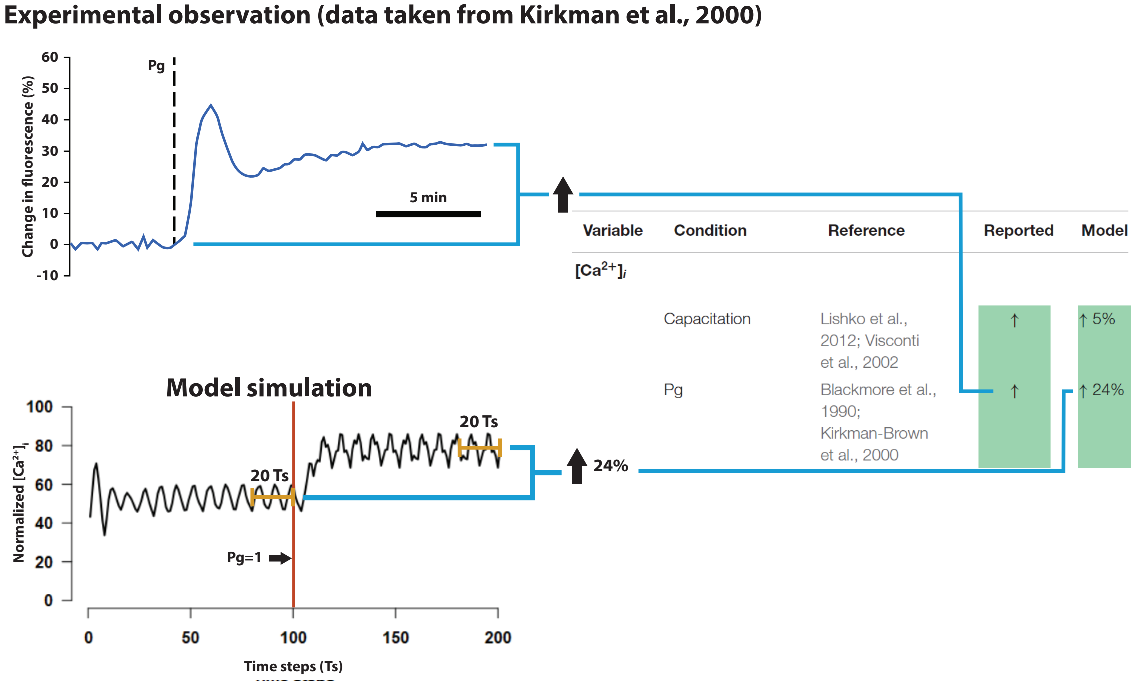

Figure S2: Comparison between model and experimental observations for $[Ca^{2+}]_i$ response to Pg stimulation. The average time series of $[Ca^{2+}]_i$ was calculated in the model before and after Pg addition. We considered the average of 20-time steps before the addition of Pg and the final 20-time steps of the simulation. Arrows indicate an increase in $[Ca^{2+}]_i$ for both the experiments and the model. The change of magnitude in the model is indicated as a percentage of the maximum possible value of $[Ca^{2+}]_i$.
